# Clinical decision-making in pediatric pneumonia: when to consider interventional: procedures? A retrospective cross-sectional study

**DOI:** 10.1097/MS9.0000000000003926

**Published:** 2025-09-17

**Authors:** Sarah Khosropanah, Hamid Kermanshahi, Leila Ameri, Seyed Javad Seyedi, Mohammad Kheirkhah, Mohsen Rahmanian, Mahdi Parvizi Mashhadi

**Affiliations:** a School of Medicine, North Khorasan University of Medical Sciences, Bojnord, Iran; b Department of General Surgery, Mashhad University of Medical Sciences, Mashhad, Iran; c Parsian Imaging Center; d Department of Pediatrics, Faculty of Medicine, Mashhad University of Medical Sciences, Mashhad, Iran; e Clinical Research Development Unit of Akbar Hospital, Mashhad University of Medical Sciences, Mashhad, Iran; f Akbar children’s hospital, Mashhad University of Medical Sciences, Mashhad, Iran; g Department of Pediatric Surgery, Faculty of Medicine, Mashhad University of Medical Sciences, Mashhad, Iran; h Division of Cardiothoracic Surgery, Cincinnati Children’s Hospital, Cincinnati, OH, USA

**Keywords:** cross-sectional study, pediatric pneumonia, predictors, surgical intervention, thrombocytopenia

## Abstract

**Background:**

Complicated community-acquired pneumonia (CCAP) in children often leads to severe complications such as pleural effusion and empyema, which may require surgical intervention. This study aimed to identify clinical, laboratory, and imaging predictors of surgical management among hospitalized pediatric pneumonia cases, including both uncomplicated and complicated presentations, to reflect the real-world clinical scenario.

**Methods:**

We conducted a cross-sectional study over 3 years (May 2020–May 2023) including 500 pediatric patients hospitalized with pneumonia. The cohort included both uncomplicated and complicated cases to capture predictors of progression to surgical intervention. Patients were categorized into those requiring surgery (*n* = 250) and those managed medically (*n* = 250). Clinical, laboratory, and imaging data were collected and analyzed using logistic regression to identify predictors of surgical intervention.

**Results:**

Key factors associated with surgical intervention included the presence of pleural effusion (odds ratio [OR] = 11.679, *P* < 0.001) and pleural peel (OR = 15.148, *P* < 0.001). Thrombocytopenia was also identified as a significant predictor (OR = 0.987, *P* = 0.005). Elevated inflammatory markers, including erythrocyte sedimentation rate and C-reactive protein, were more prevalent in the surgical group, as was a history of COVID-19, which showed a protective effect (OR = 0.153, *P* < 0.001). No significant association was found between leukocytosis or influenza history and the need for surgical management.

**Conclusions:**

Pleural effusion, pleural peel, and thrombocytopenia are key early predictors of surgical intervention in pediatric pneumonia, especially in cases progressing to CCAP. Including the full spectrum of hospitalized pneumonia cases allows for identification of prognostic factors before complications become clinically overt. These findings emphasize the importance of comprehensive assessment to guide timely surgical management. Further multicenter studies are needed to validate these predictors and improve early identification of severe pediatric pneumonia.

## Introduction

Pneumonia remains a major global cause of mortality among children under the age of 5^[1,2]^. Community-acquired pneumonia (CAP) is responsible for over 800 000 deaths of infants and young children annually. While the highest mortality rates are observed in low- and middle-income countries, CAP also significantly contributes to pediatric morbidity in high-income nations^[3]^.

**HIGHLIGHTS**

- Pleural effusion and pleural peel are the strongest predictors of surgery in pediatric CCAP.
- Thrombocytopenia is a significant marker, linked to severe disease and invasive procedures.
- Elevated ESR and CRP levels are associated with cases requiring surgical management.
- Pleural thickness shows a modest protective effect, challenging prior findings.
- Extended hospitalization reduces surgical needs by enabling intensive complication management.

Although the majority of children with CAP recover without incident, a subset may experience local pulmonary or systemic complications. Complicated community-acquired pneumonia (CCAP) is characterized by the presence of one or more local complications, including parapneumonic effusion, empyema, necrotizing pneumonia, and lung abscess^[4,5]^.

Pleural effusion represents the most common local complication of CCAP. It progresses through three distinct stages: the exudative stage (simple parapneumonic effusion), the fibrinopurulent stage (complicated parapneumonic effusion), and the organizing stage, characterized by fibroblastic activity and fibrous peel formation. The term “empyema” typically denotes the advanced stage of parapneumonic effusion and is often associated with a more severe prognosis^[4]^.

Necrotizing pneumonia, another local complication, involves extensive lung tissue destruction and liquefaction, which may occur even with appropriate antibiotic therapy^[6]^. A lung abscess, characterized by a single thick-walled cavity filled with pus due to the necrosis and suppuration of the lung parenchyma, is a rare outcome of CAP. However, lung abscesses can develop in individuals with preexisting congenital cystic lung malformations or immunodeficiencies. The progression from initial infection to abscess formation is typically gradual^[7]^.

There is no established consensus on the best approach to managing various aspects of CCAP. However, standard treatment typically involves antibiotic therapy combined with supportive care. In certain cases, interventional procedures or surgical options, such as pleural drainage or video-assisted thoracoscopic surgery (VATS), may be required to address more severe complications^[4]^. Some studies have demonstrated that VATS leads to a faster and more complete resolution of empyema than chest tube drainage alone^[8]^. However, VATS demands specialized expertise and is not widely accessible, particularly in low- and middle-income countries^[4,9]^.

Thoracotomy, which permits the removal of the thickened pleural rind and cleansing of the pleural cavity, is infrequently recommended for patients with parapneumonic effusion and empyema, because of potential drawbacks, including a large scar, increased risk of wound infection, extended recovery times, persistent air leaks, bleeding, and long-term pain^[10]^.

Chest-tube insertion, while effective for pleural infections, poses risks like pain, scarring, and failure with loculated pus. Studies suggest a conservative approach, limiting its use to severe cases (e.g., respiratory distress or sepsis), which achieves similar outcomes with fewer risks. This strategy has been linked to faster recovery markers like fever and C-reactive protein (CRP) normalization, making it a safer and equally effective alternative in managing pleural infections^[11]^.

Predicting the clinical outcomes of pediatric CAP is crucial for guiding treatment and minimizing complications. Both imaging and laboratory findings play key roles in forecasting disease severity. However, there is currently no standardized imaging technique for predicting CAP outcomes. Recent research has demonstrated a correlation between radiographic findings and the severity of CAP in children^[12,13]^. These findings include bilateral multifocal distribution, involvement of three or more sites, right hilar consolidation^[14]^, and upper lobe involvement in children over 1 year^[13]^. However, a recent study indicates that while chest X-rays can assist in evaluating hospitalized children, their effectiveness in consistently assessing pneumonia severity is limited^[1]^. Laboratory markers, including erythrocyte sedimentation rate (ESR), CRP, and leukocyte counts, might serve as valuable complements to imaging in evaluating disease severity. For example, research indicates that elevated leukocyte counts and CRP levels are strongly associated with more severe pneumonia cases, often necessitating more intensive treatments^[15]^.

This study sought to compare the clinical manifestations and imaging findings of children with complicated pneumonia who required interventional procedures or surgical intervention to those treated solely with conventional antibiotic therapy. The goal was to identify key indicators that could assist clinicians in determining when more aggressive management strategies are warranted in pediatric pneumonia.

## Materials and methods

This study has been reported in accordance with the STROCSS 2025 guidelines for cross-sectional studies in surgery^[16]^.

### Study design and participants

This analytical cross-sectional study was conducted over 3 years (1 May 2020, to 1 May 2023) and included children aged 28 days to 18 years hospitalized with pneumonia. The case group comprised all pediatric patients who underwent thoracotomy or chest tube insertion for pneumonia-related complications, confirmed through clinical presentation, radiological findings (chest X-ray), or laboratory tests (e.g., blood cultures). A census sampling method was used to include all eligible surgical cases. It is important to note that the decision to proceed with thoracotomy was influenced by the failure or limitations of VATS or thoracoscopy technology at the participating centers.

The control group included children admitted for respiratory infections (e.g., pulmonary infection, empyema, abscess, respiratory distress) who received medical management without surgical intervention. Controls were selected via simple random sampling from the same hospital population to minimize selection bias. Follow-up was conducted through institutional records and post-discharge clinic visits for at least 30 days to confirm no delayed surgical procedures.

Exclusion criteria included patients hospitalized for non-respiratory conditions and those who underwent thoracotomy or chest tube insertion for reasons unrelated to pneumonia. All patients who underwent surgical intervention initially received empirical antibiotic therapy upon admission, in accordance with the national pediatric CAP guidelines. Surgical management was pursued only in cases where clinical deterioration or lack of improvement was observed, or when radiological findings – such as increasing pleural effusion, loculation, or persistent empyema – emerged within 48 to 72 h of treatment. The average time from admission to surgical intervention was 3.8 days (range: 2–6 days), based on retrospective chart review.

### Data collection

In this study, data were collected through a researcher-designed questionnaire and reviewed from hospital archives, with quality control ensured by using standardized definitions and cross-verification by researchers. The dataset included demographic information such as patient age and gender, as well as chest imaging findings, including complications like pleural effusion and empyema. Chest ultrasound results documented the presence or absence of empyema, pleural thickening, and pleural fluid thickness, while lung computed tomography (CT) scans assessed conditions such as loculated empyema, and septa. Pleural thickening was defined as >3 mm on both ultrasound and CT imaging, based on thresholds used in previous studies and established imaging criteria in pleural pathology. Laboratory parameters gathered included complete blood count (CBC), leukocytosis (white blood cell count) with left shift, platelet count, ESR, and CRP. Clinical data, including the duration of hospitalization, history of COVID-19 or other viral infections (e.g., influenza), the occurrence of complications, readmissions, treatment success, and mortality, were recorded using a standardized checklist.

### Statistical analysis

All data were entered into R version 4.4.1 for statistical analysis. Descriptive statistics, including means, standard deviations, frequencies, and percentages, were used to summarize demographic information, clinical characteristics, and laboratory parameters.

A two-step approach was used to construct the multivariate logistic regression model. First, univariate logistic regression was performed on all candidate variables – including demographic factors (e.g., age, gender), clinical history (e.g., history of COVID-19, influenza), radiological findings (e.g., pleural thickness, pleural effusion, pleural peel), and laboratory parameters (e.g., CRP, ESR, WBC, platelet count, lymphocyte levels). Variables with a *P*-value <0.1 were considered for inclusion in the multivariate model.

The multivariate model was constructed using the enter method, allowing for simultaneous inclusion of selected variables. Odds ratios (ORs) with 95% confidence intervals (CI) were calculated to interpret the strength and direction of associations. Statistical significance was defined as *P* < 0.05.

### Ethical considerations

The study protocol was approved by the Ethics Committee under the reference code IR.MUMS.MDICSAL.REC.1402.176. The research adhered to all applicable ethical guidelines. Informed consent was obtained from all participants prior to their inclusion in the study. Questionnaire data were handled with strict confidentiality and used solely for the purposes of this study. To protect patient privacy, all identifying information was anonymized and coded before the checklists were include.

### Literature review approach

To contextualize our findings and compare them with existing evidence, we conducted a narrative literature review. Searches were performed in PubMed, Scopus, and Google Scholar databases for articles published between 2000 and 2023 in the English language.

Keywords included combinations of: pleural thickness, complicated pneumonia, pleural effusion, pediatric empyema, and surgical intervention. We prioritized studies that focused on radiological features, risk factors for surgical management, and outcomes in pediatric patients with pneumonia.

Relevant articles were screened based on title and abstract for methodological quality and alignment with the study’s objectives. Selected full-text studies were then reviewed and cited to support the interpretation and discussion of our findings

## Results

### General characteristics of the study subjects

This study included 500 children diagnosed with pneumonia, who were divided into two groups: the intervention group, consisting of 250 children who underwent thoracotomy or chest tube insertion, and the control group, comprising 250 children who received standard care. In the intervention group, 58.4% (146 children) were boys, while in the control group, 55.2% (138 children) were boys. The mean age in the intervention group was 5.93 ± 3.05 years, and in the control group, it was 6.29 ± 3.54 years. Overall, 284 participants (56.8%) were boys, and 216 (43.2%) were girls, reflecting a higher proportion of boys in the study cohort.

### Descriptive statistics of risk factor variables

The study compared clinical, radiological, and laboratory variables between pediatric patients treated surgically and those receiving medical therapy for complicated pneumonia. Significant differences were found in hospitalization length, pleural thickness, and laboratory markers, including ESR, CRP, platelet count, and lymphocyte levels (all *P* < 0.001). These results highlight key factors associated with disease severity (Table 1).

**Table 1:** Descriptive statistics of continuous variables

Variables	Mean	SD	Treatment group		Control group		*P*-value
			Mean	SD	Mean	SD	
Clinical factors							
Age	6.11	3.32	5.930	3.0682	6.290	3.5549	0.226
Length of hospitalization	9.68	6.86	13.65	7.07	5.72	3.58	<0.001*
Radiological factors							
Pleural thickness	13.22	8.13	17.05	9.425	9.41	3.809	<0.001*
Laboratory factors							
Erythrocyte sedimentation rate (mm/h)	18.52	10.75	20.3257	11.2445	16.7210	9.9485	<0.001*
C-reactive protein (mg/L)	52.20	41.06	65.7872	40.3284	38.6198	37.1730	<0.001*
Platelet	40.32	51.71	55.925	60.7292	24.7202	34.4055	<0.001*
Polymorphonuclear leukocytes (cells/L)	428.20	217.58	445.45	216.717	410.95	217.506	0.0768
Lymph	61.63	24.90	67.0916	22.8258	56.1704	25.7400	<0.001*

Moreover, the study analyzed categorical variables in pediatric patients with complicated pneumonia treated surgically or medically. Significant associations were observed for positive COVID-19 history, high Pneumonia Severity Index (PSI) scores, and the presence of pleural effusion, pleural peel, leukocytosis, and left shift (Table 2).

**Table 2:** Descriptive statistics of categorical variables

Variables	Frequency (*n*)	Percentage (%)	Treatment group			Control group		*P*-value
			Frequency		Percentage	Frequency	Percentage	
Clinical factors								
Gender (Male)	284	56.8	146	58.4		138	55.2	0.470
COVID-19 (positive history)	257	51.4	160	64		97	38.8	<0.001*
Viral infection (positive history)	76	15.2	34	13.6		42	16.8	0.32
Influenza (positive history)	100	20.0	54	21.6		46	18.4	0.372
Pneumonia Severity Index (high score)	102	20.4	87	34.8		15	6	<0.001*
Radiological factors								
Pleural effusion (presence)	198	39.6	160	64		38	15.2	<0.001*
Empyema (presence)	167	33.4	124	49.6		43	17.2	<0.001*
Pleural peel (presence)	98	19.6	93	37.2		5	2	<0.001*
Loculated empyema (presence)	32	6.4	21	8.4		11	4.4	0.0518
Septa (presence)	159	31.8	84	33.6		75	30	0.388
Laboratory factors								
Leukocytosis (presence)	120	24.0	78	31.2		42	16.8	<0.001*
Shift left (presence)	110	22.0	68	27.2		42	16.8	0.005*

### Regression analysis of thoracotomy risk factors

The multivariate analysis identified significant independent predictors distinguishing the intervention and control groups (Table 3). Key factors included hospitalization duration, history of COVID-19 infection, presence of pleural effusion, pleural thickness, pleural peel, platelet count, and lymphocyte levels (all *P* < 0.05). These findings highlight critical clinical, radiological, and laboratory indicators associated with the need for surgical intervention.

**Table 3:** Multivariate analysis of predictors between the intervention and control Groups

Variable	OR	95% CI		*P*-value
		Lower bound	Upper bound	
Clinical factors				
Hospitalization	0.677	0.615	0.745	<0.001*
PSI-Score 2	2.556	0.656	9.963	0.176
PSI-Score 3	1.535	0.507	4.646	0.448
History of infection with COVID	0.153	0.052	0.447	<0.001*
Radiological factors				
Pleural effusion	11.679	3.577	38.134	<0.001*
Empyema	2.129	0.955	4.744	0.0645
Pleural thickness	0.845	0.797	0.896	<0.001*
Pleural peel	15.148	4.082	56.210	<0.001*
Laboratory factors				
Leukocytosis	2.449	0.746	8.040	0.140
Left shift in WBC	1.204	0.356	4.075	0.765
Erythrocyte sedimentation rate (mm/h)	1.025	0.992	1.059	0.144
C-reactive protein (mg/L)	0.998	0.989	1.008	0.764
Platelet	0.987	0.978	0.996	0.005*
Lymph	0.986	0.973	1.000	0.048*

## Discussion

This study explored differences in clinical, laboratory, and radiographic features between pediatric pneumonia patients requiring surgical intervention (thoracotomy or chest tube insertion) and those managed with conventional antibiotic therapy. Our findings contribute important insights into the ongoing challenge of identifying patients with pediatric pneumonia who may require surgical intervention. By integrating clinical, laboratory, and imaging predictors, our study supports a more nuanced and early risk stratification compared to existing tools like the PSI. This approach aligns with recent research emphasizing the critical role of pleural involvement and hematological markers in guiding timely surgical decisions.

While previous studies have focused largely on isolated clinical or radiographic factors, our comprehensive analysis reflects real-world complexities and underscores the need for multidisciplinary evaluation. These insights can help clinicians optimize management strategies, potentially improving outcomes and reducing unnecessary invasive procedures. Nonetheless, prospective multicenter studies are essential to validate our findings and adapt them to diverse healthcare settings.

Hospitalization had a protective effect against the need for surgical intervention, with an OR of 0.677 (95% CI: 0.615–0.745, *P* < 0.001). Extended hospital stays allow for more intensive monitoring, timely escalation of medical therapy, and effective management of complications, reducing the progression of disease to stages that necessitate surgical intervention. For instance, a meta-analysis comparing operative and nonoperative therapies for pediatric empyema found that primary operative therapy was associated with significantly lower failure rates and shorter hospital stays compared to prolonged nonoperative management^[17]^. This underscores the role of timely and structured care in influencing outcomes and reducing the need for more invasive interventions. However, the exact mechanisms by which hospitalization achieves this protective effect warrant further investigation.

The PSI is a clinical tool widely used to predict pneumonia outcomes by scoring a range of factors, such as age, comorbidities, and laboratory findings, which together indicate the severity of pneumonia and the likelihood of complications^[18,19]^. In pediatric cases, PSI scores help guide treatment by distinguishing between mild cases that may respond to standard care and more severe cases that might require aggressive intervention^[20]^. Previous research has shown that elevated PSI scores are associated with higher mortality rates, longer hospital stays, and a greater need for intensive care in cases of pneumonia^[21]^. Despite the widespread utility of the PSI in stratifying pneumonia severity and predicting complications, our study did not find a statistically significant relationship between PSI scores and the need for thoracotomy or chest tube insertion. This outcome may be attributed to several factors that influence the clinical trajectory of complicated pediatric pneumonia.

First, while the PSI score aggregates multiple clinical and laboratory variables to estimate disease severity, it does not directly account for key radiographic findings such as pleural effusion, empyema, or pleural peel – all of which emerged in our study as strong predictors of surgical intervention. These localized complications may progress independently of the systemic factors^[5]^ considered in the PSI score, such as comorbidities or general inflammatory markers. This suggests that radiographic evaluation, particularly in the context of complicated pneumonia, may offer more direct insights into the likelihood of requiring invasive procedures. Second, the PSI’s predictive power may be more suited to guiding broad clinical management rather than determining the need for specific surgical interventions. For example, while elevated PSI scores indicate an increased risk of severe pneumonia outcomes^[22]^, they may not capture the nuanced factors that drive the decision to perform thoracotomy or chest tube insertion.

Additionally, the pediatric population poses unique challenges for the application of scoring systems like PSI, which were initially designed for adult populations^[20]^. The heterogeneity of pneumonia presentation in children – ranging from rapid resolution with antibiotics to severe complications requiring surgery^[23]^ – means that standardized tools may fail to adequately reflect the clinical nuances of younger patients. However, it is important to interpret these findings within the context of the study’s design. As a single-center, retrospective study, our results may be influenced by institutional practices, documentation variability, and selection bias. These factors could limit the generalizability of our conclusions to other healthcare settings. Nevertheless, the large sample size and comprehensive inclusion of clinical, laboratory, and radiologic variables provide a strong foundation for hypothesis generation. Future multicenter, prospective studies are needed to validate these observations and enhance external applicability.

In our study, ESR and CRP levels were elevated in cases requiring more intensive management, highlighting their role in severe pneumonia outcomes, particularly in the presence of complications like pleural effusion and empyema, though their impact was not independent. These findings align with previous research that questions their stand-alone predictive value in pediatric pneumonia^[24,25]^

The role of leukocytosis in predicting the severity of pediatric pneumonia remains debated. While leukocytosis is commonly seen in infectious and inflammatory conditions and has traditionally been regarded as a clinical marker of disease severity^[26]^, numerous studies indicate that it is a poor predictor of the underlying cause and severity of pneumonia^[27,28]^. Our results align with prior research, showing no significant association between elevated WBC counts and the requirement for intensive medical intervention, thus questioning the value of WBC levels in identifying severe infections that demand aggressive treatment. Nonetheless, it is recommended that a CBC be conducted in severe cases of CAP to help detect serious complications, such as hemolytic-uremic syndrome^[27,29]^.The significance of a left shift in leukocytosis also remains unclear in the context of pediatric pneumonia severity. Limited research exists on its association with the need for surgical interventions, and evidence linking it to disease severity remains mixed. Some studies have challenged this connection, reporting no consistent relationship between left shift and complications of pneumonia^[25]^. Our analysis similarly showed no meaningful correlation between a left shift and the requirement for invasive surgical procedures, suggesting that this marker may not reliably predict the need for surgical intervention.

Thrombocytopenia in this study was significantly associated with a higher need for procedural intervention, emphasizing its role as a marker of severe disease in CCAP. This finding aligns with the study by Sun *et al*, which demonstrated that thrombocytopenia, although less prevalent in pediatric CAP, was an independent risk factor for severe disease outcomes, including increased respiratory complications and prolonged hospital stays^[30]^. Thrombocytopenia likely reflects a heightened inflammatory response or direct platelet consumption during severe infections, which may impair hemostatic mechanisms and exacerbate disease severity^[31]^.

Additionally, prior research in adult populations has reported a significant link between thrombocytopenia and disease severity. However, to date, no studies have specifically investigated the association between platelet counts and the requirement for invasive procedures such as chest tube placement or thoracotomy. In our analysis, thrombocytopenia emerged as a significant predictor for the need for invasive interventions, with an OR of 0.987 (95% CI: 0.978–0.996, *P* = 0.005). This association between thrombocytopenia and the increased need for invasive procedures likely reflects the multifactorial role of platelets in critical illness. Thrombocytopenia, often a marker of systemic inflammation, arises in conditions such as sepsis, where platelet consumption is heightened, and production may be suppressed^[32]^. More studies are needed in the future to confirm this relationship, but it seems theoretically, the resulting hemostatic imbalance compromises vascular integrity, increasing the risk of bleeding and hematoma formation^[33,34]^, which often necessitate surgical interventions like chest tube placement or thoracotomy. Furthermore, platelets are integral to tissue repair and angiogenesis through the release of growth factors such as vascular endothelial growth factor and platelet-derived growth factor^[35]^. A deficiency in platelets may impair wound healing and exacerbate tissue damage, further increasing the likelihood of complications requiring invasive management^[36]^. Additionally, thrombocytopenia is commonly associated with advanced disease severity, serving as a surrogate marker for critical illness^[37]^.

It is also notable that studies suggesting COVID-19 as a risk factor for bacterial co-infections primarily involve adults and critically ill patients, where factors such as prolonged mechanical ventilation and intensive care settings may exacerbate bacterial superinfection risks^[38,39]^. The pediatric population may have different immune responses and exposure patterns, which could contribute to the observed protective association in our study. However, these findings warrant cautious interpretation, and larger, better-designed studies focusing on pediatric populations are needed to draw more conclusive results about the interplay between COVID-19 and bacterial coinfections.

Lymphopenia in this study was significantly associated with a higher need for procedural intervention, a finding that is both expected and consistent with previous literature. Lymphopenia often reflects an impaired adaptive immune response, which can exacerbate the severity of infections and reduce the body’s ability to effectively combat pathogens. In the context of CCAP, reduced lymphocyte levels may indicate a compromised immune system unable to control inflammation and bacterial proliferation, leading to more severe disease states such as pleural effusion or empyema^[40]^.

In this study, we found significant differences in pleural involvement between children with pneumonia who required interventional procedures and those managed with medical therapy alone. The incidence of pleural effusion was markedly higher in the intervention group, affecting 64% of patients compared to only 15.2% in the control group. This difference was highly significant (*P* < 0.001), and the analysis further confirmed pleural effusion as a key independent predictor of the need for surgical intervention, with an OR of 11.679 (95% CI: 3.577–38.134, *P* < 0.001). These findings align with published research that identifies pleural effusion as a direct indication for surgical intervention when conservative treatments prove insufficient^[41]^.In pediatric pneumonia, pleural effusion or empyema does not always necessitate surgical intervention, as antibiotic therapy alone can often be effective. However, delaying drainage for more than 7 days has been linked to increased mortality^[42]^. Studies by Islam *et al* and Hilliard *et al* indicate that when pleural effusion progresses to a complicated parapneumonic effusion or empyema, the likelihood of requiring interventional procedures increases markedly^[8,10]^. Unlike pleural effusion, empyema did not emerge as a significant independent predictor for surgical intervention in our analysis. This lack of statistical significance implies that while empyema is indeed linked to complications in pediatric pneumonia, it may not solely drive the need for invasive procedures when other factors are considered. This finding aligns with existing research, including a recent study by Daniel Lichtenstein *et al*, which highlights certain characteristics – such as younger age, increased empyema fluid density, pleural thickening, and fluid locations – serve as stronger predictors of surgical necessity in pediatric empyema secondary to pneumonia^[43]^.

This study highlights pleural peel as a significant predictor of the need for surgical intervention in pediatric pneumonia cases. Pleural peel was present in 37.2% of the intervention group, contrasting with only 2% in the control group, a statistically significant difference (*P* < 0.001). The development of pleural peel is part of the organizing stage of pleural effusion, where ongoing inflammation leads to the formation of thickened fibrous tissue along the pleura^[44]^. This tissue layer limits lung expansion and hinders effective antibiotic delivery, often rendering conservative treatment inadequate^[45]^. Notably, among patients with pleural empyema or pleural peel who were managed conservatively, clinical improvement was consistently observed during hospitalization, and no delayed surgical interventions were required during follow-up.

While pleural effusion was included in our analysis as a binary variable, pleural thickness served as a pragmatic proxy for estimating effusion burden, given the lack of consistent volumetric measurements. Unlike prior research^[45]^, our study found that pleural thickness demonstrated a modest protective effect against the need for surgical intervention, with an OR of 0.845 (95% CI: 0.797–0.896, *P* < 0.001)(49,52). This unexpected finding may be attributed to differences in study design and analytical approaches. In particular, earlier studies often excluded key variables, such as clinical and laboratory factors and pleural peel – a radiological feature strongly associated with surgical intervention – from their analyses. By omitting these factors, prior research may have overestimated the predictive value of pleural thickness alone. Additionally, methodological variations, such as differences in statistical modeling, sample sizes, or population characteristics, could have contributed to the observed discrepancies. For example, our inclusion of a comprehensive set of variables and a large sample size allowed for a more nuanced assessment of pleural thickness in the context of other influential factors, potentially explaining its modest protective effect in our study. These findings highlight the need for standardized methodologies in future research to better clarify the role of pleural thickness in predicting surgical outcomes.

In our analysis, pleural thickening was defined as >3 mm on both ultrasound and CT. Although pediatric-specific thresholds for significant pleural thickening are not uniformly established in the literature, this cutoff reflects prior studies that have used similar criteria. For example, Jaffe *et al* incorporated >2 mm pleural enhancement in a pediatric CT severity score for empyema, while Copley *et al* and Wang *et al* considered pleural thickness >3 mm to be indicative of significant pathology. Given that the normal pleura measures <0.5 mm in healthy children, even mild thickening can represent a pathologic process. Our threshold aligns with these observations and offers a practical, reproducible benchmark for radiological assessment^[46,47]^.

This study’s single-center design limits its generalizability, as variations in management practices and patient demographics across regions may affect the applicability of the findings. The retrospective nature of the data collection introduces potential biases, particularly related to incomplete or inconsistent clinical and radiological documentation. Future research should include multicenter, prospective studies to validate these findings. Exploring long-term outcomes postsurgical intervention and improving access to minimally invasive techniques such as VATS is critical for optimizing the management of complicated pediatric pneumonia. Additionally, some important confounding variables – such as preexisting comorbidities, prior antibiotic usage, and time since symptom onset – were not consistently documented and thus could not be included in our analysis. The absence of these variables introduces the possibility of residual confounding. Future prospective studies should aim to incorporate these factors to further clarify their impact on surgical outcomes in complicated pediatric pneumonia.

Pleural fluid analysis was not included in our model due to inconsistent documentation and limited availability. In many instances, cytological and biochemical data were either absent or incomplete in the retrospective records. To maintain data consistency, this variable was excluded from multivariate analysis. Similarly, although thoracentesis is a minimally invasive therapeutic option often considered before surgical intervention, its use and outcomes were inconsistently recorded in our dataset. This precluded its evaluation as a distinct management approach. Future prospective studies should aim to incorporate pleural fluid characteristics and systematically assess the role of thoracentesis as key diagnostic and therapeutic factors in pediatric CCAP.

Furthermore, the 30-day follow-up period was designed to capture short-term outcomes and the immediate need for surgical intervention. However, this may be insufficient for certain conditions – for example, pleural peel managed conservatively – where delayed progression or subsequent indications for surgery may occur well beyond the initial month. Because pleural peel represents the organizing phase of empyema and can evolve slowly^[48]^, extended observation would provide a more complete understanding of its natural history, treatment response, and optimal timing for surgical intervention. Future prospective studies should therefore incorporate longer follow-up intervals to address this gap.

## Conclusion

This study underscores the critical clinical, radiological, and laboratory factors linked to severe pediatric pneumonia necessitating surgical intervention. Key indicators, such as pleural effusion and pleural peel, were closely associated with increased disease severity, highlighting the importance of prompt recognition and intervention. Thrombocytopenia emerged as a significant marker of severe disease, correlating with a higher likelihood of requiring surgical intervention, while leukocytosis showed no meaningful association with the need for chest intubation or thoracotomy. These findings emphasize the complexity of managing severe pediatric pneumonia and the necessity of integrating clinical, radiological, and laboratory data for comprehensive evaluation. Further research should aim to validate these predictors and develop strategies to enhance outcomes through early detection and individualized treatment approaches.

## Data Availability

Dataset isn’t publicly available.

## Research registration unique identifying number (UIN)

This study was retrospectively registered with ResearchRegistry.com (Unique ID: researchregistry10895). The registry entry is available here.
